# 2D Materials Based Optoelectronic Memory: Convergence of Electronic Memory and Optical Sensor

**DOI:** 10.34133/2019/9490413

**Published:** 2019-08-22

**Authors:** Feichi Zhou, Jiewei Chen, Xiaoming Tao, Xinran Wang, Yang Chai

**Affiliations:** ^1^Department of Applied Physics, The Hong Kong Polytechnic University, Hong Kong; ^2^Institute of Textiles and Clothing, The Hong Kong Polytechnic University, Hong Kong; ^3^School of Electronic Science and Engineering, Nanjing University, Nanjing, China

## Abstract

The continuous development of electron devices towards the trend of “More than Moore” requires functional diversification that can collect data (sensors) and store (memories) and process (computing units) information. Considering the large occupation proportion of image data in both data center and edge devices, a device integration with optical sensing and data storage and processing is highly demanded for future energy-efficient and miniaturized electronic system. Two-dimensional (2D) materials and their heterostructures have exhibited broadband photoresponse and high photoresponsivity in the configuration of optical sensors and showed fast switching speed, multi-bit data storage, and large ON/OFF ratio in memory devices. In addition, its ultrathin body thickness and transfer process at low temperature allow 2D materials to be heterogeneously integrated with other existing materials system. In this paper, we overview the state-of-the-art optoelectronic random-access memories (ORAMs) based on 2D materials, as well as ORAM synaptic devices and their applications in neural network and image processing. The ORAM devices potentially enable direct storage/processing of sensory data from external environment. We also provide perspectives on possible directions of other neuromorphic sensor design (*e.g*., auditory and olfactory) based on 2D materials towards the future smart electronic systems for artificial intelligence.

## 1. Introduction

The advance of digital technology enables data storage and processing in binary form with high speed, accuracy and density. The development of computing hardware has been driven by Moore's law in the past few decades. However, the downward scaling trend is hitting the scaling limits according to the International Technology Roadmap for Semiconductors (ITRS) 2.0. Different from the strategy of device miniaturization involved in the digital components, a new trend of “More-than-Moore” (MtM) is highlighted in the ITRS 2.0, which is featured as a diversified function of data collection and information storage/ processing devices. The function diversification described in MtM can be considered as another form of “scaling down” for the electronic system, which includes noncomputing functions into the digital ones, enabling the interaction of digital devices with the outside world directly. Device integration of noncomputing (e.g., sensors and actuators) and digital components (e.g., memories and computing units) is one of the main approaches for function diversification of MtM. Considering the large occupation proportion of image data in both data center and edge devices, we will mainly discuss the integrations of optical sensors and digital devices (data storage and processing) in this review.

Optoelectronic random-access memories (ORAMs) offer great opportunities for multifunctional integration of optical sensing, data storage and processing into one single device. In contrast to traditional random-access memories (RAMs), optical excitation provides another method to manipulate the memory cell in ORAMs, which enables to store light information with an electronic readout at low programming voltages and is beneficial for the storage and transport of secured information. The combination of both optical stimuli and electrical readout voltage has potential for enlarging the ratio of low resistance state (LRS) and high resistance state (HRS), thus enabling multilevel data storage and improving data storage capacity. From another aspect, the optical trigger is employed in ORAM for the switching, bringing the benefits of wide bandwidth and reduction of electrical loss during sensory data transmission [1–6]. In addition, it is promising to perform digital processing functions with light as an extra control parameter by combining both optical and electrical inputs as input signals, such as arithmetic and logic operations and coincidence detection [4, 7]. Furthermore, the integration of optical sensors and memory devices in ORAMs provides possibilities in simplifying the image sensor circuitry.

**Figure 1:**
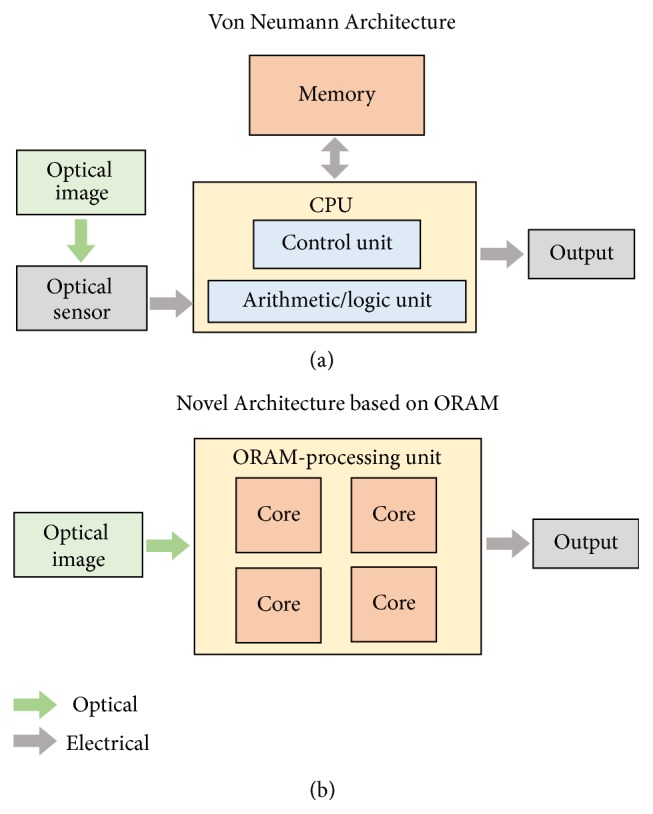
(a) Illustration of conventional Von Neumann architecture for image processing. (b) Novel computing architecture based on ORAM/ORAM synaptic devices for image processing.

In the conventional von Neumann computing architecture, memory and processing units are physically separated, which is inefficient in terms of energy consumption and data transmission speed. The human brain consists of a large collection of neurons connected through synapses, and deals with learning and processing complex cognition tasks (*e.g.,* pattern recognition, and multiobject detection) with high energy efficiency. The power consumption in human brain is 5 orders magnitude lower than conventional computers [8]. Neuromorphic computing mimics the working schemes of information storage and processing like the human brain. The electronic memory synaptic devices are the most investigated and fundamental component in the hardware-based neuromorphic computing. The tunable resistance under continuous stimuli of voltage or current inputs is similar to the synaptic weight (generally determined as biological strength of the connection between two neurons). However, the conventional memory synaptic devices for neuromorphic computing are manipulated through the electrical method and are isolated from the sensors (e.g., visual, auditory or olfactory) [9–17]. ORAM synaptic devices integrate optical sensors and synaptic RAMs, which can respond to optical stimuli and exhibit light-tunable synaptic behaviors, including light-tunable short-term plasticity (STP), long-term plasticity (LTP), spike-timing-dependent plasticity (STDP), and spatiotemporal learning rule (STLR). The development of ORAM synaptic devices can provide potentials for bridging the gap between optical (visual) sensing and neuromorphic computing [18].  Figure 1 illustrates a comparison of the conventional Von Neumann architecture and a computing architecture based on the ORAM/ORAM synaptic devices for image storage and processing. The architecture based on ORAM/ORAM synaptic devices offer the possibilities of directly storing and processing the optical information, in contrast to the separated memory and processing unit and preliminary optical-to-electrical signal conversion for image processing in the conventional Von Neumann architecture.

Among different materials systems for ORAMs, two-dimensional (2D) materials, such as graphene, transition metal dichalcogenides (TMDs) (including MoS_2_, WS_2_, WSe_2_, MoSe_2_, MoTe_2_,* etc*.) and black phosphorus, have exhibited unprecedented electrical and optical properties for the applications of soft optoelectronics and nonvolatile memories. In terms of the integration of optical sensing, data storage and processing, 2D materials and their heterostructures also exhibit promising potentials for building ORAMs with broadband photo response, excellent photoresponsivity [19–25], fast switching speed, multibit data storage, large ON/OFF ratio and high energy efficiency [26–32]. More specifically, (1) the strong light-matter interactions and the gate tunability owing to the unique physical and structural properties in the ultrathin 2D materials benefit the construction of both volatile and nonvolatile ORAMs. (2) The large-surface-to-volume ratio in these 2D materials allows significant photo-excited charge trapping and further the ORAM design. (3) The mechanical flexibility and optical transparency of 2D materials are appropriate for the application of ORAMs in an artificial eye. The structure and physical properties of 2D materials have been overviewed in the existing reports [33–35]. In this paper, we mainly focused on the review and perspective of 2D materials for memory applications.

## 2. 2D Materials Based Nonvolatile Electronic Memory

The 2D materials based electronic memory provides ideas for the design of 2D materials based ORAM structure. The working mechanisms of state-of-the-art ORAMs are generally based on charging trapping in three-terminal FET memory and light-tunable filament formation/rupture in two-terminal resistive random-access memories (RRAMs). In this part, we will mainly review two types of nonvolatile electronic memory based on 2D materials: floating gate memories and RRAMs.

**Figure 2:**
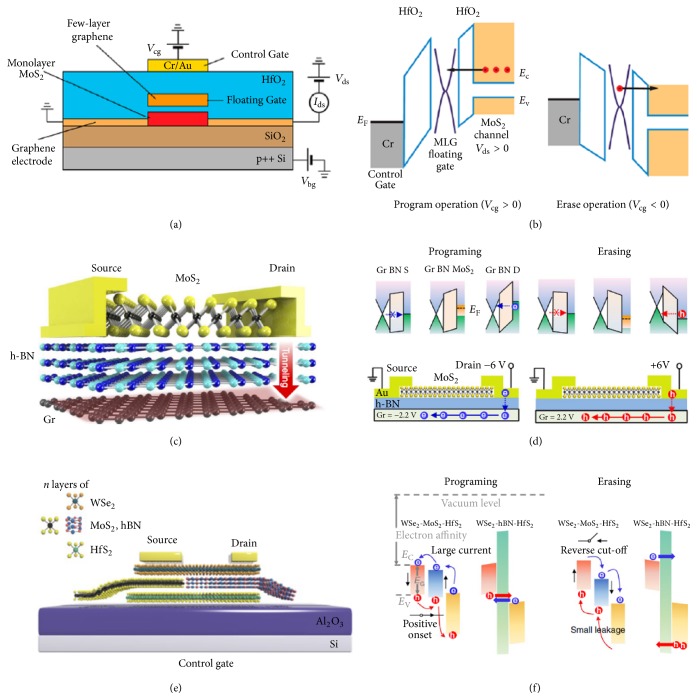
Floating gate memory based on 2D materials. (a) Schematics and (b) band alignments of programming and erasing processes of a floating-gate memory based on MoS_2_/graphene heterostructure. (c) Schematics and (d) Band alignments of programming and erasing processes of a two-terminal floating gate memory based on MoS_2_/h-BN/graphene heterostructure. (e) Schematics and (f) band alignments of programing and erasing processes of a semifloating gate memory. Reproduced with permissions: (a) and (b) from [36]; (c) and (d) from [37]; (e) and (f) from [28].

2D materials and their heterostructures have offered great practical advantages in the design of floating gate memory device with a large ON/OFF ratio and long retention time. The application of 2D materials as the floating gate can effectively reduce the capacitive interference between the floating gate and drain electrode for high-capacity data storage. In early 2013, Bertolazzi et al. designed a nonvolatile floating gate memory with MoS_2_ as channel material, multilayer graphene as floating gate and charge trapping layer, and HfO_2_ as control gate (Figure 2(a)). As a positive control gate voltage is applied (+18 V), electrons tunnel from MoS_2_ channel to multilayer graphene floating gate through the HfO_2_ tunneling barrier. The electrons are stored in the floating gate, programming the device. A negative control gate voltage (-18 V) sweeps the electrons in the floating gate back to the channel material, erasing the device (Figure 2(b)). With this structure, the device exhibits nonvolatile switching characteristics with ON/OFF ratio of 10^4^ [36]. Later, Choi et al. demonstrated a similar floating gate memory with MoS_2_/h-BN/graphene heterostructure with h-BN as a tunneling barrier, showing a comparable ON/OFF ratio of 10^4^ [37]. By further tuning the h-BN thickness appropriately, two-terminal operation of the MoS_2_/h-BN/graphene floating gate memory can be achieved (Figure 2(c)), which allows for higher integration density. A source-drain voltage of -6 V programs the device to LRS state by driving the electrons to tunnel through the h-BN layer and be accumulated at graphene floating gate. A positive source-drain voltage of 6 V then is employed for erasing the device (Figure 2(d)). The device exhibits an extremely low OFF current of 10^−14^ A, large ON/OFF ratio of up to 10^9^, and long retention time (10^4^ s) [38]. As the operation speed in floating gate memories is usually relatively slow, a semi-floating gate memory based on van der Waals heterostructures was further developed as an effective device structure to achieve ultrahigh speed and long refresh time (Figure 2(e)). In the semi-floating gate memory, WSe_2_ is the channel material. HfS_2_ and h-BN serve as a floating gate and tunneling layer (drain region), respectively. The WSe_2_/MoS_2_ (source region) works as a* p-n* junction switch. For the programming operation (V_g_ = − 5 V, V_DS_= 0 V), the* p–n* junction is switched on. The electrons in the HfS_2_ are transferred to the channel material through the h-BN tunneling layer (flash path) or the WSe_2_/MoS_2_* p–n* junction (switch path). For the erasing operation (V_g_ = 5 V, V_DS_= 0 V), as the* p–n* junction is switched off, electrons in the channel are transferred to the floating gate and combined with holes in the HfS_2_ layer. The fast writing speed and long refresh time in the device design originate from the fast charge injection through switch path and floating gate charge storage, respectively [28].

**Figure 3:**
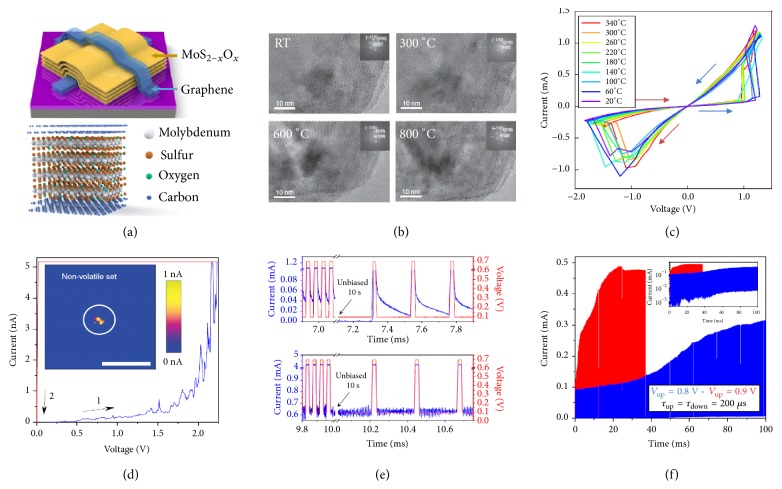
Two-terminal RRAM devices based on 2D materials. (a) Schematic of the graphene/MoS_2−x_O_x_/ graphene device. (b) HRTEM images of MoS_2−x_O_x_ at different temperatures. MoS_2−x_O_x_ still maintains a well-defined crystal structure at 800°C. (c) I-V switching curves of the graphene/ MoS_2−x_O_x_/graphene device at different temperatures. (d) C-AFM test that reveals the formation of conductive filament in Au/Ti/ h-BN/Cu synapse. (e) and (f) Dynamic responses of Metal/ h-BN/ metal synapses with different programming methods. Reproduced with permissions: (a), (b), and (c) from [29]; (d), (e), and (f) from [39].

Although excellent nonvolatile properties can be achieved in the 2D nonvolatile floating gate memory, its three-terminal structure limits the integration density and requires large programming voltage. Thus, the two-terminal RRAMs are considered as promising candidates towards nonvolatile memory applications for the high integration density and low programming voltage. Two-terminal RRAMs based on 2D TMD monolayers have also been rationally designed for low operation voltage, including MoS_2_, MoSe_2_, WS_2_ and WSe_2_ [32]. The good thermal stability and well-defined interfaces of 2D heterostructures provide possibilities in overcoming the thermal stability issue in the conventional RRAMs. Wang* et al.* reported a robust RRAM based on graphene/MoS_2−x_O_x_/graphene heterostructure with excellent endurance up to 10^7^ switching cycles and high operating temperature of 340°C (Figures 3(a) and 3(c)) [29]. The high-temperature endurance of the device originates from the atomically sharp interfaces even at elevated temperatures, avoiding the undesirable ion migrations (Figure 3(b)). The 2D materials RRAMs also show promising potentials in constructing artificial synapses with low power consumption for energy-efficient neuromorphic computing. Shi* et al.* reported a 2D material RRAM synaptic device with metal/h-BN/metal structure (Figure 3(d)) based on the switching mechanism of formation/rupture of conductive filament (~15 nm). The RRAM synaptic device exhibits both nonvolatile and volatile switching behaviors, as well as the tunable synaptic behaviors (Figures 3(e) and 3(f)). The low power consumption (0.1 fW in standby and 600 pW per transition) and fast switching speed (10 ns) implicate the potential applications for brain-like computing [39].

## 3. 2D Materials Based Nonvolatile ORAM

The conventional 2D materials based RRAM is usually manipulated by electrical triggers. As 2D materials have exhibited a broad response from ultraviolet to infrared light with high responsibility or fast response speed, it is possible to integrate the optical sensing and data storage together by rationally designing device structures, which is potential for the various applications, such as image sensors for artificial vision, optical demodulators and logic gates for optoelectronic circuits, and synaptic devices for neuromorphic systems. The constructions of ORAMs can be mainly divided into two types according to the device structure: ORAM based on 2D materials and ORAM based on 2D heterostructures, which will be discussed in the Sections 3.2 and 3.3, respectively.

### 3.1. Nonvolatile ORAM* versus* Photodetectors

**Figure 4:**
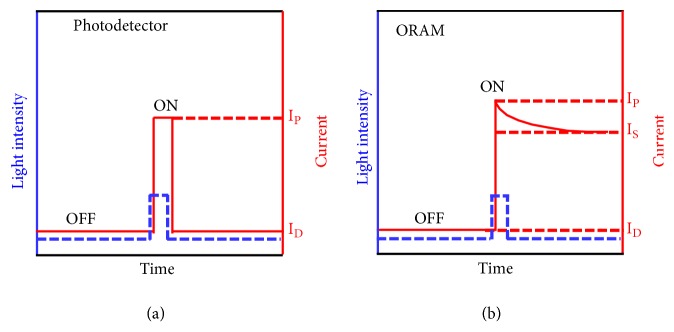
Comparison of photo-responses of (a) conventional photodetectors and (b) ORAMs.

Both ORAMs and traditional photodetectors convert the optical signal to an electronic one. However, the design of ORAMs is in sharp contrast to the photodetectors, in which the photo-generated charge carriers recombine immediately in the photodetectors after the removal of light stimuli, corresponding to an immediate current drop from *I*_*p*_ (photo current) to *I*_*D*_ (dark current) (Figure 4(a)). In ORAM, the optical information can be stored and retained for a long period even after the removal of light stimuli, corresponding to a stored and retained photocurrent *I*_*S*_ (Figure 4(b)). The responsivity R in photodetector, which represents the capability of optical-to-electrical conversion, can be expressed according to

(1) $$\begin{array}{c} R\left(\;A/W\;\right)=\frac{I_{P}-I_{D}}{P\times S} \end{array}$$

where *P* is the light power density and *S* is the device area. As the photocurrent *I*_*p*_ usually suffers from a slight degradation (after the removal of light stimuli) until reaching a stable current storage current of *I*_*S*_, here we can define a specific nonvolatile responsivity (NR) for ORAM in a similar way, which can be expressed as

(2) $$\begin{array}{c} NR\left(\;A/W\;\right)=\frac{I_{S}-I_{D}}{P\times S} \end{array}$$

where *I*_*S*_ is the storage current.

### 3.2. 2D Materials Based ORAM

The working mechanism of ORAMs based on 2D materials mainly lies in providing charge trapping centers for photo-generated charge carriers (electrons/holes), which store the photo-generated electrons/holes even after the removal of light stimuli. In this case, the optical information can be stored in the ORAM. Because the light intensity determines the amount of photo-generated charge carriers, which is generally related to the possibility of charge trapping or interfacial physics. Thus, tuning the light intensity provides an approach to achieve multiple resistance states. Additionally, light stimuli with different wavelengths correspond to different optical excitation energies and different amount of light absorption, which may also induce to different charge trapping/detrapping physics and multiple resistance states.

**Figure 5:**
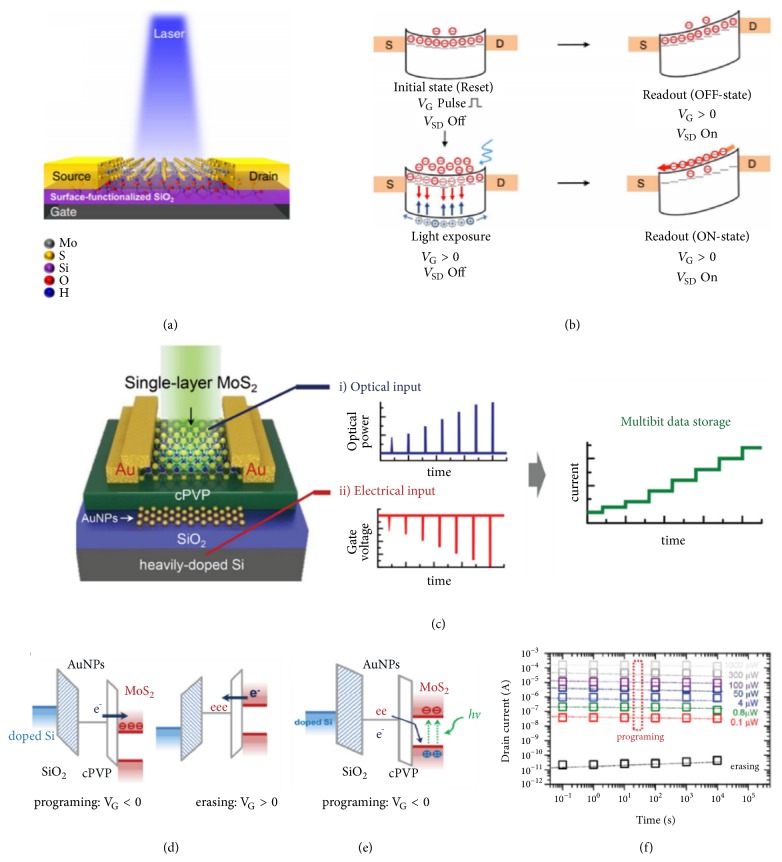
(a) Schematic illustration of MoS_2_ ORAM with functionalized MoS_2_/SiO_2_ interface. (b) Band diagrams of the initial state, upon light exposure and reading process. (c) Schematic illustration of MoS_2_ ORAM with Au nanoparticles as charge trapping layer. (d) Band diagram of electrically-controlled programming and erasing. (e) Band diagram of light-controlled programming. (f) Light-controlled multilevel resistance states. Reprodued with permissions: (a) and (b) from [41]; (c), (d), (e), and (f) from [42].

In 2014, Lei et al. demonstrated multilayered CuIn_7_Se_11_ based ORAM array for image sensing, and further extended to other atomically thin 2D materials (InSe and MoS_2_) [40]. The design concept of the ORAM is to build potential well with an energy band bending between 2D material channel and metal electrodes, trapping photo-generated electrons. Although the ORAM exhibits good photo-response, its short retention time (less than 50 s) and low ON/OFF ratio (less than 10) limit the applications for multilevel nonvolatile memories. Further, Lee et al. introduced artificially charge traps at the MoS_2_/SiO_2_ interface through O_2_ plasma treatment of SiO_2_ before MoS_2_ monolayer growth (Figure 5(a)). The band alignments of the initial state, upon light exposure and reading process for ON and OFF states are illustrated in Figure 5(b). At the initial state, a potential well is formed by metal-semiconductor Schottky barrier with the applied gate voltage. A positive gate voltage pulse first programs the device to OFF state by driving the electrons in the channel to the interfacial trap states, leading to a low OFF current of 4 pA (V_sd_=3V). Upon light illumination (450 nm laser), photo-generated electrons are accumulated in the potential well, while holes escape from both electrodes through upward energy band bending. After the removal of light illumination, the readout process (readout bias is applied) eliminates one of the Schottky barriers and releases the stored electrons. By intentionally introducing charge traps, the MoS_2_ based ORAM device exhibits improved ON/OFF ratio of 4700 and retention time of 6000 s. However, the photo-current still suffers from relatively large degradation after the removal of light exposure [41].

Towards the design of ORAMs with high photo-responsivity, large ON/OFF ratio, low dark current and good retention properties, researchers adopted other charge trapping methods to fabricate multilevel and highly responsive nonvolatile 2D ORAM. Cho group successfully reported a multibit MoS_2_ ORAM with high ON/OFF ratio of 10^7^, long retention time over 10^4^ s, and high nonvolatile responsivity exceeding 8000 A/W at an illumination power of 0.1 *μ*W [42]. They employed a layer of Au nanoparticles as the charge trapping layer and floating gate between the blocking and tunneling dielectrics to store photo-generated charge carriers in the MoS_2_ ORAM (Figure 5(a)). The MoS_2_ ORAM can be programmed in both electrical and optical manner, which enables versatile applications, such as performing logic operations and coincidence detection [3]. The schematic band diagrams of electrical and optical programming are illustrated in Figures 5(d) and 5(e), respectively. For the electrical programming, a negative gate voltage induces the transfer of electrons in Au nanoparticle layer to the conduction band of MoS_2_ layer through Fowler-Nordheim tunneling; for the optical programming, the electrons can be easily transferred from Au nanoparticle layer to MoS_2_ valence band with the light illumination (655 nm laser), which prevents the recombination of photo-generated electrons and holes in the MoS_2_ layer. The programmed resistance states can be erased by applying a positive gate voltage. The MoS_2_ ORAM exhibits light intensity-dependent multilevel resistance states, integrating both optical sensing and nonvolatile data storage (Figure 5(f)).

**Figure 6:**
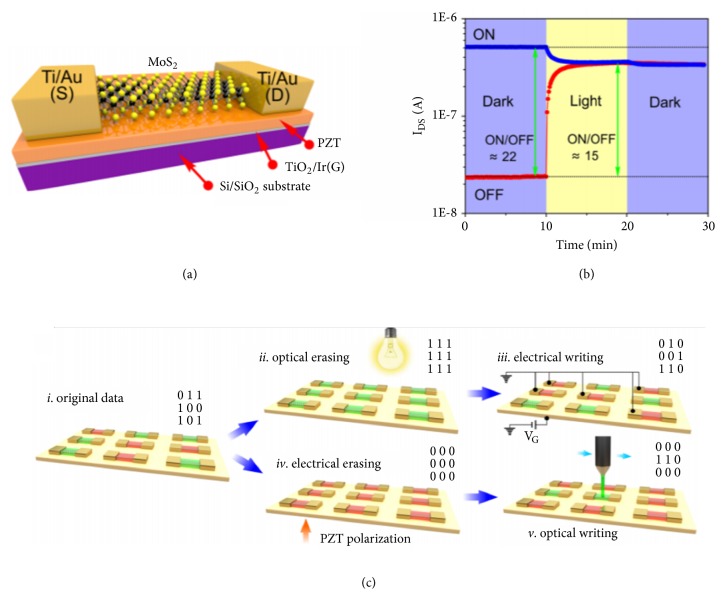
(a) Schematic of MoS_2_ ferroelectric ORAM. (b) Retention characteristics after the removal of light stimuli. (c) Global operations of “optical erase and electrical write” and “electrical erase and optical write.” Reproduced with permission from [43].

In addition to the typical FET ORAMs, ferroelectric ORAM based on monolayer MoS_2_ has also exhibited unique characteristics, including nondestructive data readout and low operating voltage [43] (Figures 6(a) and 6(b)). The optical illumination can induce the domain re-arrangement in the MoS_2_-PZT ORAM, which further causes the change in conductivity. The ferroelectric ORAM can be globally optically SET and electrically RESET or electrically SET and optically RESET, depending on the initial polarization states of PZT (Figure 6(c)). Wang* et al.* fabricated a two-terminal MoS_2_ ORAM with a structure of Au/MoS_2_ nanospheres/Au [44]. With a poling voltage of 6 V, upon the light illumination the device can be switched from HRS to LRS at -2.9 V with an ON/OFF ratio of 10. A positive voltage of 4.2 V can switch the device back to HRS. By combining different poling voltage and light illumination, multilevel resistance states can be obtained in the two-terminal MoS_2_ ORAM device, which provides possibilities to the design of two-terminal ORAM based on 2D materials.

### 3.3. 2D Heterostructure Based Nonvolatile ORAM

The 2D material based ORAMs are generally sensitive to environmental factors because most of the memory functions in ORAMs are dependent on the charge trapping carriers in original or artificial defects at the materials interface. The charge trapping mechanism usually leads to relatively short retention time in the memory device. By combining the 2D materials with other semiconductors, the ORAMs based on 2D heterostructure can widen ORAM design possibilities and improve the ORAM performance, including the light absorption range, environmental stability and responsivity, nonvolatile characteristics and data storage capability.

**Figure 7:**
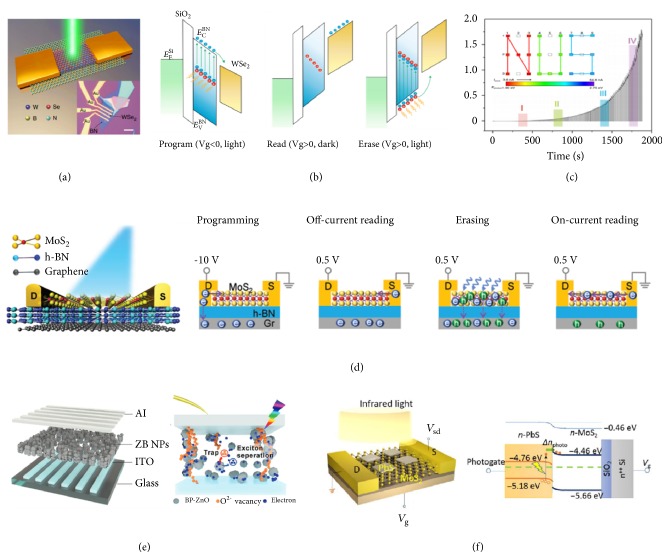
(a) Schematic structure and (b) schematic band diagrams of switching mechanism in WSe_2_/h-BN ORAM. (c) 130 storage states obtained by applying a different number of 405 nm light pulse (duration of 0.5 s and intensity of 210 mW/cm^2^). The gate voltage is -80 V. Inset: integrated WSe_2_/h-BN pixel matrix for color image sensor with memory capability. Two-terminal ORAMs based on different heterostructures. (d) Schematics of ORAM with MoS_2_/h-BN/graphene heterostructures and illustration of programming, erasing and ON/OFF readout processes. (e) Schematics of ORAM with phosphorene/ZnO nanoparticles heterostructure and illustrations of the SET process with external bias and light illumination. (f) Schematic of MoS_2_/PbS infrared ORAM and band diagram of MoS_2_/PbS heterostructure upon the infrared laser illumination. Reprodued with permissions: (a), (b), and (c) from [46]; (d) from [47]; (e) from [48]; (f) from [49].

Roy et al. reported an ORAM based on graphene/MoS_2_ vertical heterostructure, which demonstrates a high photo-responsivity of 5×10^8^ A/W at room temperature. However, the switching ON/OFF ratio is less than 2, owing to the high electrical conductivity of graphene and the large dark current of the device. The low ON/OFF ratio restricts the application for multibit ORAM [45]. Later, Du et al. designed a broadband (ultraviolet to visible region) ORAM based on WSe_2_/h-BN heterostructure (Figure 7(a)), which exhibits a large ON/OFF ratio of 1.1×10^6^, over 128 distinct resistance states and remarkable retention time over 4.5×10^4^ s [46]. Upon the light illumination (405 nm laser), the photo-generated electrons in BN conduction band can be transferred to WSe_2_ conduction band under a negative gate voltage of -20 V (programming process). The photo-generated holes are left and localized in the middle of BN bandgap even after the removal of light stimulation and electric field, which indicates the nonvolatile switching characteristics. A positive gate voltage with light illumination erases the device and turns the device back to the initial states by driving holes from BN to WSe_2_ (Figure 7(b)). Figure 7(c) illustrates 130 resistance states obtained by applying an increased number of light pulses. The WSe_2_/h-BN can respond to the lights with a wide range of wavelength from 410 nm to 750 nm. A color image sensor array integrated with sensing and storage capability exhibits promising potentials for the future design of smart image sensor (Figure 7(c) inset). The heterostructure configuration can also be extended to other materials, such as BP/ BN based ORAM, which exhibits ultrahigh nonvolatile responsivity of 1.2×10^7^ A/W [46]. However, the three-terminal FET ORAM usually involves with high programming gate voltage, relatively low integration level and complex circuitry for integration. Two-terminal ORAM is highly desirable for the design of the device with simple structure, high integration level and low power consumption.

To further decrease the programming voltage and power consumption in ORAM device, Tran* et al. *demonstrated a nonvolatile multibit ORAM based on MoS_2_/BN/graphene van der Waals heterostructure, showing a programming voltage (source-drain voltage) of -10 V, extremely low dark-current of 10^−14^ A and high nonvolatile responsivity of 2×10^4^ A/W (@458 nm, 2nW) (Figure 7(c)) [47]. A negative source-drain voltage of -10 V induces the electrons tunneling through BN to graphene floating gate. The accumulated electrons in the graphene layer serve as a negative gate bias, thus programs the device with extremely low dark current (LRS). The visible light pulses then induce the photo-generated holes in MoS_2_ layer, tunneling through the low hole barrier of MoS_2_/BN and combining with the stored electrons in the graphene layer, and RESET the device to HRS (Figure 7(d)). This operation scheme leads to a high ON/OFF ratio of 10^6^ and over 16 distinct storage levels by tuning the light intensities [47]. Hu* et al.* reported a two-terminal and vertical ORAM based on BP/ZnO nanoparticles heterojunction, exhibiting broadband absorption from ultraviolet (380 nm) to near infrared (785 nm) and low SET voltage < 4V (Figure 7(e)).The optically-controlled resistive switching originates from fast electron/hole separation in the heterojunction, which is further involved in the manipulation of the formation/rupture of oxygen vacancy filament [48].

Most of the absorption spectrum in ORAM device currently is limited in the ultraviolet and visible region. As infrared radiation is the communication medium for data transfer in wireless networks, night vision, image sensing, medical diagnosis, etc., the extension of absorption to the infrared region in ORAM is highly desirable, which not only enables the conversion and storage of infrared data, but also benefits for the optical data communication in the photonic circuit and artificial visual intelligence. The application of 2D heterostructures provides possibilities in designing infrared ORAMs by combining 2D materials with small bandgap semiconductors or up-conversion nanoparticles. Wang et al. reported a nonvolatile infrared ORAM by adopting a MoS_2_/PbS van der Waals heterostructure, which can respond to a wide wavelength range from 880 nm to 1940 nm (Figure 7(f)) [49]. The optical switching originates from the localized photo-generated holes in PbS near the interface and the injection of photo-generated electrons from PbS to MoS_2_, thus modulating the MoS_2_ conductivity. A retention time of over 10^4^ s and multi-level storage states can be obtained by applying 1940 nm infrared light pulses. The nonvolatile responsivity reaches up to 1.2×10^4^ A/W. The up-conversion nanoparticles based on lanthanide ions-doped NaYF_4_ with narrow absorption band at 980 nm also provide possibilities for 2D materials based infrared ORAM. The up-conversion nanoparticles/MoS_2_ serve as charge trapping and separation centers upon the infrared light illumination, which is responsible for the optical assisted switching process [50]. The up-conversion nanoparticles/MoS_2_ based infrared ORAM enables to sense and store the 980 nm infrared light with different light intensities, corresponding to different resistance states.

Table 1 compares the optical switching characteristics of nonvolatile ORAMs based on 2D materials and their heterostructures with other material systems. The nonvolatile ORAMs based on 2D materials have exhibited high responsivity, low optical power consumption, large ON/OFF ratio, good endurance, and broadband absorption compared with that based on other materials (e.g., oxides, hybrid organic-inorganic perovskite, organic materials), however, the high programming voltage in most of 2D material ORAM is required to be further lowered to achieve a low power consumption in the future.

## 4. 2D Materials Based ORAM Synaptic Device

### 4.1. ORAM Synaptic Device versus Photodetector

**Figure 8:**
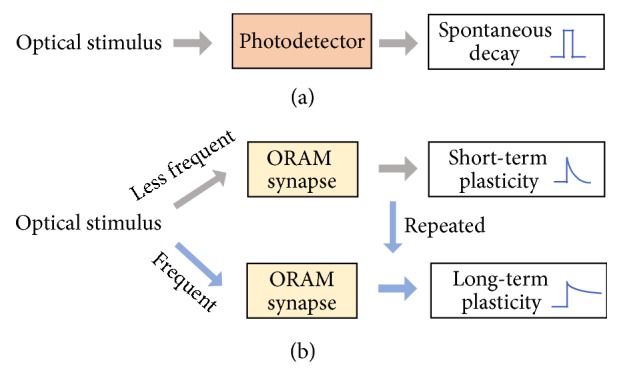
Comparison of photo-responses of (a) photodetectors without tunable plasticity and (b) ORAM synaptic device with light-tunable plasticity.

The integration of optical sensing and data storage in ORAM enables direct storage of optical information from the outside world without optical-to-electrical conversion, however, these functions are restricted in the digital applications. Towards the future low power neuromorphic computing, ORAM synaptic devices are desirable that provide possibilities to bridge the sensing and neuromorphic computing and the direct processing of optical information (e.g., image processing) [18]. In comparison to conventional photodetectors and nonvolatile ORAMs, ORAM synaptic devices exhibit both optically-controlled nonvolatile and volatile switching characteristics. It can respond to optical stimuli and exhibit light-tunable synaptic behaviors, such as STP, LTP and STDP.  Figure 8 illustrates the light modulating schemes in photodetectors (a) and ORAM synaptic devices (b). In sharp contrast to the conventional photodetector or image sensors, ORAM synaptic devices sense information in ways like neural signals with light-tunable and time-dependent plasticity, implicating the potential application in the construction of neuromorphic visual systems.

### 4.2. ORRAM Synaptic Device

**Figure 9:**
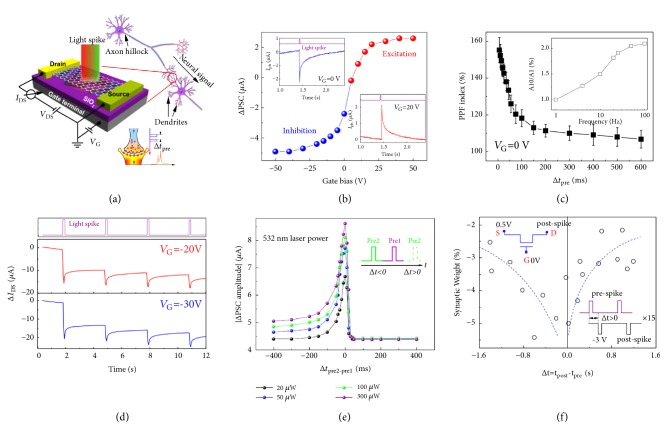
(a) Schematics of graphene/CNT based ORRAM synaptic device. (b) STP: post-synaptic current stimulated with an optical pulse at various gate bias. (c) PPF index as a function of pulse interval. (d) LTP obtained by combining optical pulse and voltage pulse. (e) Illustration of STLR learning rule. (f) Illustration of STDP learning rule. Reproduced with permission from [54].

Qin* et al. *first reported an ORAM synaptic device based on graphene/carbon nanotube (CNT) heterostructure, demonstrating light-tunable synaptic behaviors, such as, light-tunable STP, LTP, STLR and STDP (Figure 9(a)) [54]. The channel conductance can be tuned through optical pulse and gate pulse. The multiple state conductance can be regarded as the biological synaptic weight. Figure 9(b) shows the STP stimulated with an optical pulse (405 nm, 50 *μ*W, 5 ms), where the conductance increases abruptly and relaxes within seconds. The STP is triggered owing to the electron transfer from CNT to graphene upon the light illumination, tuning the built-in field formed at graphene/CNT interface. Optically tuned paired-pulse facilitation (PPF) function is an important function for temporal visual information encoding, as illustrated in Figure 9(c). The plasticity can be further modulated to LTP by combining a gate voltage and an optical pulse (Figure 9(d)). The LTP originates from the trapping of photo-generated holes from graphene or SWCNT at the SiO_2_ surface owing to the defects. A STDP learning rule (symmetric STDP) is also demonstrated in this work (Figure 9(f)), which is related to associative learning and enables to the application of coincidence detector for spiking activity. Both the non-Hebbian learning rule (e.g., STLR) and Hebbian learning rule (*e.g.,* STDP) are mimicked (Figures 9(e) and 7(f)), respectively. The implementations of STLR and STDP are associated with the spatiotemporal information processing in the neural network. Similar STLR and STDP learning rules can also be performed in ion-gated 2D ORAMs [55, 56].

Other types of 2D materials heterostructures have also been utilized for ORAM synaptic devices, such as MoS_2_/perylene-3,4,9,10-tetracarboxylic dianhydride (PTCDA) heterojunction, exhibiting large synaptic weight change and PPF ratio [22]. To further broaden the absorption spectrum and reduce the power consumption, a hybrid structure of 2D WSe_2_/boron-doped Si nanocrystals was employed for ORAM synaptic device with wide absorption spectrum from ultraviolet to near-infrared and low power consumption of 75 fJ, which points the way to the development of low-power artificial visual intelligence beyond human eyes [57].  Table 2 compares the basic performances of ORAM synaptic devices based on 2D materials with other different active layers.

### 4.3. Applications in Neural Network and Image Processing

**Figure 10:**
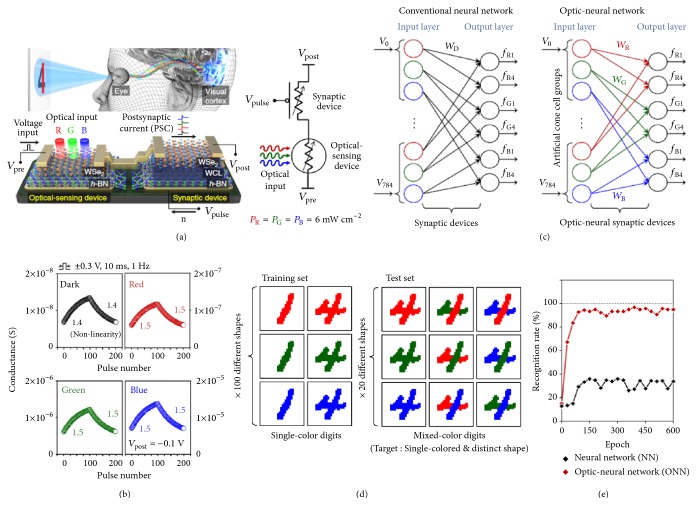
(a) Schematics of device structure and interconnection. (b) Wavelength-dependent conductance change for long-term potentiation and long-term depression. (c) Comparisons of conventional NN and ONN. In conventional NN, the synapses cannot sense the optical information. In ONN, optical sensing functions are added to the synaptic devices, exhibiting wavelength-dependent synaptic weights. (d) Training set and test set for the color-mixed pattern recognition. (e) Comparisons of recognition rate as a function of training epochs in NN and ONN. Reproduced with permission from [64].

The multiple synaptic functions demonstrated in the ORAM synaptic device facilitate the coupling of optical sensing and neuromorphic computing, and enable the applications in the optical neural network for image processing (*e.g.,* pattern recognition). Generally, for the conventional image processing based on neuromorphic computing, the optical signals in the images are first converted to electronic signals through image sensors. The electronic signals are then transferred to the synaptic arrays in the neural network for further processing. In contrast to the conventional memory synaptic devices operated by electrical method, the ORAM synaptic devices respond to the optical stimuli directly and exhibit light-evoked synaptic behaviors. The constructed optic-neural network through ORAM synaptic devices can potentially simplify the circuitry for neuromorphic image processing and reduce the power consumption during the data transmission. Additionally, the high integration level in ORAM synaptic device provides future miniaturized and power-efficient artificial eye design [18]. Tian* et al. *first developed an optic-neural network (ONN) with optical synapses based on graphene/2D perovskite, for which the pattern recognition rate in this ONN can reach up to 80 % [58]. Seo* et al.* further demonstrated an ONN for mimicking color-mixed pattern recognition in human vision. The ORAM synaptic device is integrated with a WSe_2_/h-BN photodetector and a synaptic device based on WSe_2_/weight control layer (WCL)/h-BN (Figure 10). The optical synapse can respond to the lights with different wavelengths (red, green and blue), showing different synaptic weight change for long-term potentiation/long-term depression (Figure 10(b)). According to the wavelength-dependent synaptic weight change, an ONN (Figure 10(c)) is developed for the task of color-mixed pattern recognition, which differentiates from the conventional neural network (NN) without wavelength-dependent synaptic weight control. The recognition rate for mixed-color digits (Figure 10(d)) in ONN is around 60% higher than that in NN [64].

## 5. Conclusions and Perspectives: 2D Materials in Designing Neuromorphic Devices

Owing to the strong light-matter interaction, unique physical properties, electrostatic tunability and downward scaling potential, 2D materials and their heterostructures have been widely employed for optoelectronics and memory applications in recent years. Towards the trend of functional diversification in “MtM”, the integration of optical sensing, data storage and processing drives the design of ORAM and optoelectronic synaptic devices, which can potentially simplify the circuit complexity and reduce the power consumption for the photonic circuit and artificial vision. In contrast to the conventional image sensors with light-intensity sensing, the ORAM device with light tunable plasticity (light-dosage sensing) can further be applied for the construction of ONN for pattern recognition, potentially bridging the sensory data and neural network in the neuromorphic visual system. The nonvolatile ORAMs based on 2D materials heterostructures have exhibited high photoresponsivity, stable multilevel data storage and good retention and endurance properties. Although ORAM devices based on 2D materials have exhibited various advantages over other materials, the high programming voltage is still a main issue, which causes high power consumption. Additionally, the three-terminal structure in most of the 2D material ORAMs restricts the integration density in the integrated circuits. To develop appropriate 2D material ORAMs with two-terminal structure can be a potential approach for low-power and high-integration-density devices in the future.

The ORAM synaptic device that integrates optical sensing and synaptic functions have exhibits great potentials in neuromorphic computing and visual information processing. For the future artificial intelligence, to mimic the sensing and processing of auditory or olfactory information like the human brain is also demanded for energy-efficient and miniaturized processing system. As the conventional visual, olfactory and auditory sensors generate a large amount of redundant data, neuromorphic sensory devices potentially allow for the real-time storage and processing of the sensory data with reduced power consumption. Similar to the extension from optical sensors to ORAM synaptic devices, olfactory sensors (e.g., gas sensors) and the auditory sensors (e.g*.,* sound detectors) can also be potentially developed as neuromorphic sensors with synaptic behaviors through appropriate structure design. For both sound detectors and gas sensors, 2D materials have also exhibited their potentials in achieving high sensitivity. More specifically, 2D materials (TMDs, BP, silicene, MXene, etc.) are adopted as promising candidates for high-sensitive gas sensors because of the large surface-to-volume ratio and tunable functionality of the surface through decorating transition metals or functional groups [65–69]. The excellent mechanical properties and piezoelectric properties in 2D materials also implicate the applications of sound detectors [70–72]. The outstanding performances in 2D based gas sensors and sound detectors suggest the possibilities for future designs of neuromorphic olfactory sensors and auditory sensors, which could contribute to important applications in the development of edge computing and Internet of Things.

## Figures and Tables

**Table 1:** Comparisons of ORAMs based on 2D materials with other material systems.

Active layer	Three /two terminal	Control gate/floating gate	Switching mechanism	Light wavelength (nm)	Light intensity (*μ*W cm^−2^)	Light pulse width (ms)	Non-volatile responsivity (A/W)	Max On/off	Retention (s)	Programming voltage (V)	Multi-level	Enduran-ce	Ref.
graphene/MoS_2_	Three	SiO_2_/-* *-	Charge trapping	635 nm, white light	37.6	30-100	-* *-	<2	10^5^	50	Yes	-* *-	[45]

MoS_2_	Three	Functionalized SiO_2_/-* *-	Charge trapping	450, 650 nm	0.019 *μ*W	1-3000	-* *-	4700	10^4^	80	Yes	-* *-	[41]

CuIn_7_Se_11_	Three	SiO_2_/-* *-	Charge trapping	543 nm	1.5×10^5^	50-500	-* *-	<10	50	80	-* *-	-* *-	[40]

MoS_2_/PVP/AuNPs	Three	SiO_2_/AuNPs	Charge trapping	655 nm	0.1-1000 *μ*W	1000	8000	10^7^	10^4^	10	Yes	200	[42]

MoS_2_/PZT	Three	SiO_2_/-* *-	Ferroelectric	halogen bulb	150 W	-* *-	-* *-	22	10^4^	6	-* *-	500	[43]

WSe_2_/BN	Three	SiO_2_/-* *-	Charge trapping	410-750 nm	2 nW	500	-* *-	10^6^	4.5×10^4^	20	Yes (128)	200	[46]

BP/BN	Three	SiO_2_/-* *-	Charge trapping	410-500 nm	2 nW	500	1.2×10^7^	415	4×10^4^	15	Yes	200	[46]

MoS_2_/h-BN/graphene	Two	-* *-/graphene	Charge trapping	458,638, 725, 811 nm	2nW-160nW	10-2000	2×10^4^ @458 nm@2nW	10^6^	4.5×10^4^	10	Yes (18)	10^4^	[47]

BP/ZnO	Two	-* *-	Oxygen filament	380, 532, 633, 785 nm	20-500	-* *-	-* *-	10^6^	-* *-	5	-* *-	-* *-	[48]

MoS_2_/PbS	Three	SiO_2_/-* *-	Charge trapping	850, 1310, 1550 nm	27	1000	1.2×10^4^@1940 nm	150	10^4^	No programming voltage required	Yes	2000	[49]

MoS_2_/NaYF_4_:Yb^3+^, Er^3+^	Two	-* *-	Charge trapping	980 nm	0-5×10^5^	-* *-	-* *-	10^4^	10^4^	0.5	Yes	200	[50]

CH_3_NH_3_PbI_3-x_Cl_x_	Two	-* *-	Charge trapping	White light	0-3200	1000	1×10^3^	10^4^	10^4^	0.1	Yes	400	[3]

CeO_2-*x*_ /AlO_y_	Two	-* *-	Charge trapping	499, 560, 638 nm	400	20000	1	30	10^4^	No programming voltage required	Yes	30	[4]

ZnO nanorods/Nb-doped SrTiO_3_	Two	-* *-	Charge trapping	365 nm	2.5×10^6^	-* *-	1.3	10^3^	10^4^	2	-* *-	-* *-	[51]

ROT300/VOTc/ZnO nanparticles	Three	Al/ZnO nanparticles	Charge trapping	850 nm	80-10500	1000	-* *-	10^4^	10^6^	No programming voltage required	Yes	-* *-	[52]

BBTNDT	Three	SiO_2_/-* *-	Charge trapping	White light	40000	10000	433	10^6^	10^6^	120	-* *-	-* *-	[53]

**Table 2:** Comparisons of ORAM synaptic devices based on 2D materials with other material systems.

Active layer	Three /two terminal	Operation mechanism	Spike wavelength	Spike energy (pJ)	Ref.
graphene/CNT	Three	Charge trapping	405, 532 nm	250	[54]
Si-NC/WSe_2_	Three	Charge trapping	375, 532 and 1342 nm	0.000075	[57]
MoS_2_/PTCDA	Three	Charge trapping	532 nm	5.38	[22]
2D Perovskite/graphene	Three	-* *-	520 nm	0.592	*[58]*
CH_3_NH_3_PbI_3_	Two	Charge trapping	500, 635 nm and halogen bulb	-* *-	*[2]*
CdS/CNT	Two	Charge trapping	405 and 532 nm	-* *-	[59]
InGaZnO_x_/Al_2_O_3_	Three	Charge trapping	365 nm	2.4×10^3^	[60]
Pentacene/PMMA/CsPbBr_3_ quantum dots	Three	Charge trapping	365, 450, 520 and 660 nm	0.76	[61]
ITO/Nb:SrTiO_3_	Two	Charge trapping	459, 528 and 630 nm	1178	[62]
C8-BTBT	Three	Charge trapping	360 nm	2160	[63]
